# A 12‐session relapse prevention program vs psychoeducation in the treatment of Japanese alcoholic patients: A randomized controlled trial

**DOI:** 10.1002/npr2.12248

**Published:** 2022-03-27

**Authors:** Takayuki Harada, Yuzo Aikawa, Mihoko Takahama, Yosuke Yumoto, Mitsuru Umeno, Yukako Hasegawa, Shigeo Ohsawa, Nozomu Asukai

**Affiliations:** ^1^ 13121 Faculty of Human Sciences University of Tsukuba Tokyo Japan; ^2^ Saitama Prefectural Psychiatric Hospital Saitama Japan; ^3^ Hanwa Izumi Hospital Osaka Japan; ^4^ National Hospital Organization Kurihama Medical and Addiction Center Kanagawa Japan; ^5^ APARI Clinic Tokyo Japan; ^6^ Tokyo Metropolitan Institute of Medical Science Tokyo Japan; ^7^ Aoki Hospital Tokyo Japan

**Keywords:** alcoholic patient, alcoholism, cognitive behavioral therapy, psychoeducation, randomized controlled trial, relapse prevention

## Abstract

**Aim:**

Alcoholism is the most prevalent substance use disorder in Japan; the estimated number of patients and high‐risk drinkers is in the millions. Although studies in the West have shown that cognitive behavioral therapy (CBT) is one of the most effective treatment strategies for alcoholic patients, there is a dearth of efficacy studies of CBT‐based intervention for those patients in the non‐Western setting. The aim of this study is to investigate the efficacy of a 12‐session CBT‐based relapse prevention program for Japanese alcoholic patients.

**Methods:**

Forty‐eight alcoholic patients (M = 36, F = 12) who were admitted to an addiction treatment unit were randomly allocated either to a 12‐session relapse prevention (RP) program (n = 24) or a 12‐session psychoeducation (PE) program (n = 24). Both treatment programs were conducted in a group format once a week for 12 weeks. Other aspects of inpatient treatment (group meetings, etc) were the same in both groups. Self‐rating scales, which measure behavioral and cognitive coping, coping response, self‐efficacy, and cognition of drinking, were administered at pretreatment, mid‐treatment, and posttreatment periods. The proportion of participants who relapsed at 3 and 6 months after discharge was evaluated.

**Results:**

Both RP and PE groups showed significant improvement in self‐efficacy and cognition of drinking at posttreatment. However, there were no significant differences in the self‐rating scales between both groups. In addition, there were no significant differences in relapse rate at 3 and 6 months after discharge between both groups.

**Conclusions:**

The 12‐session CBT‐based relapse prevention program and the psychoeducation program may be equally efficacious for alcoholic patients. Several factors that influenced the results are discussed.

## 1 INTRODUCTION

Alcohol dependence is one of the most prevalent public health concerns in Japan. In Japan, it is estimated that more than 4.5 million people are considered to be problem drinkers and 800,000 meet the criteria for alcohol dependence of *the ICD‐10 Classification of Mental and Behavioral Disorders*.^1^ However, only 40,000 patients are currently under treatment, and the most common treatment approach, except for self‐help groups, is pharmacologic, while cognitive‐behavioral or relapse prevention strategies are rarely used.^2^

Previous studies have shown that cognitive‐behavioral therapy (CBT) is one of the most effective treatment strategies for alcoholic patients.^3^ From the CBT perspective, alcoholism is viewed as not only a disease but also a set of learned behaviors.^4^ For example, alcoholic patients are likely to use alcohol to alleviate their negative emotions. Through repeated experiences where alcohol works to provide the desired effects, drinking becomes the only way to achieve them. Moreover, in the learning process, originally neutral stimuli are associated with drinking and they work as triggers to drinking.

The relapse prevention (RP) model is a CBT‐based treatment approach targeting substance abuse and addictive behaviors. The major goal of RP is to address the problem of relapse and to generate techniques for preventing or managing its occurrence.^5^ RP has two major steps: (a) identifying high‐risk situations or triggers and (b) learning coping skills for these triggers in order to prevent relapse. RP incorporates a variety of treatment elements to prevent relapse and achieve abstinence, including coping skills training, anger management, stress management, and cognitive restructuring. Sandahl and Rönnberg^6^ developed a group format for RP and showed its effectiveness for patients with alcohol dependence. Irvin et al^7^ conducted a meta‐analytic review of RP and found that the overall effect size was *r* = .14 (95% confidence interval [CI] = 0.10‐0.17). A larger effect size was obtained (*r* = .48, 95% CI = 0.42‐0.53) when psychosocial functioning was used as an outcome. According to another recent meta‐analysis^8^ of the efficacy of CBT for adult alcohol and illicit drug users, CBT produced a small but statistically significant treatment effect (*g* = 0.15, 95% CI = 0.07‐0.24). However, these studies were carried out exclusively in the United States. Furthermore, RP has rarely been conducted outside Western countries.^9^

The aim of this study is to investigate the efficacy of a CBT‐based relapse prevention program for Japanese alcoholic patients compared to treatment as usual, which includes psychoeducation. Although psychoeducation (PE) has been one of the core components as well as pharmacological therapy in the treatment of Japanese alcoholic patients, its effectiveness as an active treatment also has not been evaluated in Japan. Therefore, this study also gave us an opportunity to compare the effectiveness between those two active treatments, RP and PE. As far as we know, this study is the first randomized controlled trial of a CBT‐based relapse prevention program for alcoholic patients in Japan.

## 2 METHODS

### 2.1 Participants

Participants in this study were recruited from patients who were admitted to the addiction treatment unit of the Tokyo Metropolitan Matsuzawa Hospital. The recruitment period was from August 2011 to March 2012. The inclusion criteria were (a) 20 years of age or above, (b) patients who had an ICD‐10 diagnosis of alcohol dependence (F10.2), and (c) patients who were able to respond to a self‐administered questionnaire. The exclusion criteria were (a) patients who had a psychotic disorder as a primary diagnosis (schizophrenia and other psychotic disorders, mood disorders with psychotic features) and (b) patients who had serious medical or neurological complications. The diagnosis of psychotic disorder was made by psychiatrists (YA, MT, and YY) using the Mini‐International Neuropsychiatric Interview (M.I.N.I.).^10^, ^11^

Among 49 eligible patients, no patients were excluded, but one patient refused to participate in the study, so the total number of participants was 48. All participants were physically detoxified from alcohol prior to their involvement in the study. At pretreatment, we assessed the severity of alcohol dependence using the Alcohol Use Disorders Identification Test (AUDIT)^12^, ^13^, ^14^, ^15^, ^16^, ^17^ and comorbid mental health disorders using the M.I.N.I.

We obtained written informed consent from all participants prior to their participation. This study was approved by the Institutional Review Boards of the Tokyo Metropolitan Matsuzawa Hospital and the Tokyo Metropolitan Institute of Medical Science (approval number H23‐03). Our trial protocol was registered at the University Hospital Medical Information Network Clinical Trials Registry (UMIN‐CTR) (http://www.umin.ac.jp/ctr/index.htm).

### 2.2 Randomization

Participants were randomly assigned either to the relapse prevention (RP) program or the psychoeducation (PE) control group. An independent research assistant who was not involved in recruitment and treatment conducted randomization using computer‐generated random digit numbers. In randomization, a block design with the size of 4 and 6 was used to minimize the possibility of a chance imbalance of sample size between the groups. Each group had 24 participants. The proportion of women was significantly less in the RP group than in the PE group (4.2% vs 45.8%, *χ* ^2^(1) = 12.57, *P* < .01), and the mean AUDIT score was significantly lower in the RP group than in the PE group (23.3 vs 27.8, *t*(23) = 2.05, *P* < .05). There was no significant difference in other variables between the groups at baseline (Table 1).

**TABLE 1:** Demographic data of participants

Variables	Treatment groups		*P*
	Relapse prevention (n = 24)	Psycho education (n = 24)	
Mean age (SD)	53.3 (9.2)	49.0 (12.9)	.190
Gender, n (%)**			
Men	23 (95.8)	13 (54.2)	<.001**
Women	1 (4.2)	11 (45.8)	
Marital status, n (%)			
Unmarried/Divorced	18 (75.0)	17 (70.8)	.7453
Married	6 (25.0)	7 (29.2)	
Employment, n (%)			
Employed	7 (29.2)	7 (29.2)	1.000
Unemployed	17 (70.8)	17 (70.8)	
Education, n (%)			
Under college‐level education	16 (66.7)	16 (66.7)	1.000
College‐level education	8 (33.3)	8 (33.3)	
History of prior psychiatric hospitalization, n (%)	14 (58.3)	12 (50.0)	.562
Drug abuse, n (%)	4 (16.7)	3 (12.5)	.683
Criminal history, n (%)	7 (29.2)	5 (20.8)	.505
AUDIT mean score (SD)*	23.3 (8.8)	27.8 (6.4)	.049*

Abbreviation: AUDIT, Alcohol Use Disorders Identification Test.

*P < .05, **P < .001.

### 2.3 Intervention

The duration of admission in our addiction treatment unit is usually 3 months, and during admission, all participants in both groups were offered the weekly scheduled program: group meetings, physical exercises, occupational therapy (leatherwork), and a family session. The patients were encouraged to attend Alcoholics Anonymous meetings during the inpatient period and to present their drinking history in a group meeting before discharge. Participants had either relapse prevention (RP group) or psychoeducation (PE group) once a week for 12 weeks in an open group format. Each group had 8‐15 participants and each session for both groups was 90 minutes in duration. As the program was delivered continuously, participants who entered at different points of time still covered the full range of topics despite their staggered entry into the program. After discharge, participants were encouraged to receive continuous outpatient treatment.

#### 2.3.1 CBT‐based relapse prevention (RP)

The RP group used a workbook, which consisted of 12 sessions covering self‐management to prevent relapse, including identification of drinking triggers, coping skills training, cognitive restructuring, stress management, anger management, and alternative activities. Moreover, considering Japanese cultural and social characteristics, culturally appropriate components were incorporated including culturally appropriate drinking triggers and coping skills.^2^ Facilitators of RP sessions included a psychiatrist, an occupational therapist, and two clinical psychologists. We also developed a fidelity scale for this study and treatment fidelity was checked by at least two staff members.

#### 2.3.2 Psychoeducation (PE)

Our psychoeducation program consisted of 4 weekly 90‐minute sessions, which included the following topics: mechanism and psychological characteristics of addiction, relationships with family members, harmful consequences of addiction, and a roadmap to recovery. The PE group received three cycles of this four‐session program repeatedly, for a total of 12 sessions. The program was conducted by two psychiatrists and a clinical psychologist.

### 2.4 Measurements

To evaluate psychological factors related to treatment effects and relapse, the following four self‐rating scales were administered at three points of time: pretreatment, mid‐treatment (immediately after the sixth session), and posttreatment.

#### 2.4.1 Coping Behaviors Inventory (CBI)

The CBI^18^ is designed to assess the use of behavioral and cognitive coping strategies of alcoholic patients to cope with craving and high‐risk situations. It is a 4‐point scale and respondents indicate the frequency of coping behaviors from 0 (usually tried this) to 3 (never tried this). The validity and reliability have been evaluated by several studies.^18^, ^19^

#### 2.4.2 Drug Abuse Self‐efficacy Scale

This scale^20^ is used to evaluate self‐efficacy to cope with craving. It is comprised of two subscales: generalized self‐efficacy and situation‐specific self‐efficacy. The former uses a 5‐point scale and the latter a 7‐point scale. Good reliability and validity of the scale have been confirmed in Japanese samples.^20^

#### 2.4.3 Tri‐Axial Coping Scale (TAC‐24)

Kamimura et al^21^ developed the Tri‐Axial Coping Model, which categorized coping responses into three axes: problem‐focused vs emotion‐focused, approach vs avoidant, and behavior‐oriented vs cognition‐oriented. The TAC‐24 was designed to assess variation in the coping repertoire based on this model and it has shown good reliability and validity.^21^ The scale consists of 20 items in eight subscales. Each item is measured with a 5‐point rating.

#### 2.4.4 Drinking‐Related Cognitions Scale (DRCS)

The DRCS^22^ is designed to assess multidimensional cognitions of drinking, including perception of drinking problems, perception of impaired drinking control, readiness to change, decisional balance, and self‐efficacy in alcoholics. The scale is comprised of 15 items with 6‐point ratings. Good reliability and validity of the scale have been confirmed.^22^

### 2.5 Primary and secondary outcomes

The primary outcome of the treatment in this study was a self‐report on relapse at 3 and 6 months after discharge. Relapse was defined as drinking an amount of alcohol that was equal to or greater than that before admission. The secondary outcome was the change in scores between pre‐ and posttreatment on the four self‐rating scales.

### 2.6 Data analyses

We performed the chi‐square test to compare the relapse rates between groups at 3‐ and 6‐month follow‐up assessments. For the intent‐to‐treat analysis, we used the general linear model (GLM) repeated measures procedure using scale scores as dependent variables, [time] (pre‐, mid‐, and posttreatment) as the within‐subject variable, [group] as the intersubject variable, and [time × group] interaction. Pairwise comparison was adjusted with Bonferroni's method. Considering the significant difference in gender ratio between the groups, we also performed the same GLM analysis for a subgroup of male participants. The last observation carried forward method for missing data from participants who dropped out was utilized in the ITT analysis.

## 3 RESULTS

Among the 48 participants, 75% were men and the mean age was 51.0 years (standard deviation [SD] 11.3; age range, 27‐68 years). With regard to educational attainments, 33.3% had the college level education. Of the total, 27.1% were married, 39.6% were single, and 33.3% were divorced. In addition, 29.2% were employed. More than half had a prior history of admission to a psychiatric hospital for alcohol dependence (54.2%).

**FIGURE 1:**
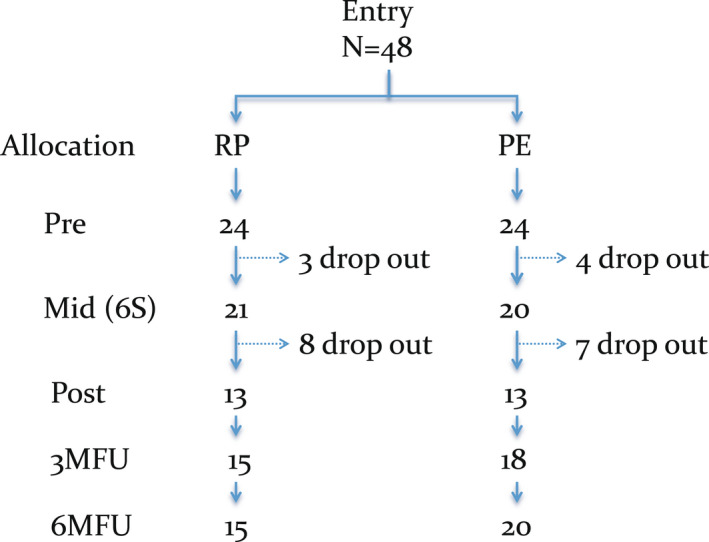
Flow of participants through the study. PE, psychoeducation group; RP, relapse prevention group

Of the 48 participants, three in the RP group and four in the PE group dropped out by the midpoint assessment after completion of the sixth session. Among those, one in the RP group committed suicide and another in the PE group developed acute psychosis. Furthermore, eight in the RP group and seven in the PE group dropped out between the midpoint and the final 12th session. Therefore, 13 participants in each group completed the program and there was no difference in drop‐out rates between the groups. We obtained follow‐up data from 15 participants in the RP group and 18 in the PE group at 3 months after discharge, and 15 in the RP group and 20 in the PE group at 6 months after discharge (Figure 1).

### 3.1 Primary outcome

Relapse rate at 3 months (*χ* ^2^(1) = 0.004, *P* = .948) and 6 months (*χ* ^2^(1) = 0.380, *P* = .537) after discharge did not differ significantly between the groups (Table 2).

**TABLE 2:** Outcome measures at 3‐ and 6‐mo follow‐up after discharge (ITT analysis)

	3‐mo follow‐up			6‐mo follow‐up		
	RP (n = 15)	PE (n = 18)	*P*	RP (n = 15)	PE (n = 20)	*P*
Relapse, %	40.0	38.9	.948	40.0	30.0	.537
CBI(SD)	51.0 (18.1)	50.0 (17.0)	.871	46.4 (13.4)	47.0 (17.3)	.912
SE (SD)	73.7 (20.2)	76.7 (20.0)	.672	77.1 (20.1)	75.2 (18.4)	.587
TAC‐24 (SD)	70.2 (11.7)	73.5 (11.9)	.430	71.3 (11.5)	73.4 (11.0)	.782
DRCS (SD)	59.7 (12.6)	65.6 (10.5)	.152	61.3 (14.0)	66.9 (11.0)	.194
Drop‐out, %	12.5	16.7	.683	54.1	54.1	1.000

Note

Relapse is defined as drinking amount of alcohol that was equal of grater that that before admission.

Abbreviations: CBI, Coping Behaviors Inventory; DRCS, Drinking‐Related Cognitions Scale; PE, psychoeducation group; RP, relapse prevention group; SD, standard deviation; SE, Self‐Efficacy Scale; Self‐efficacy, drug abuse self‐efficacy scale; TAC‐24, Tri‐axial Coping Scale‐24.

### 3.2 Secondary outcomes

**FIGURE 2:**
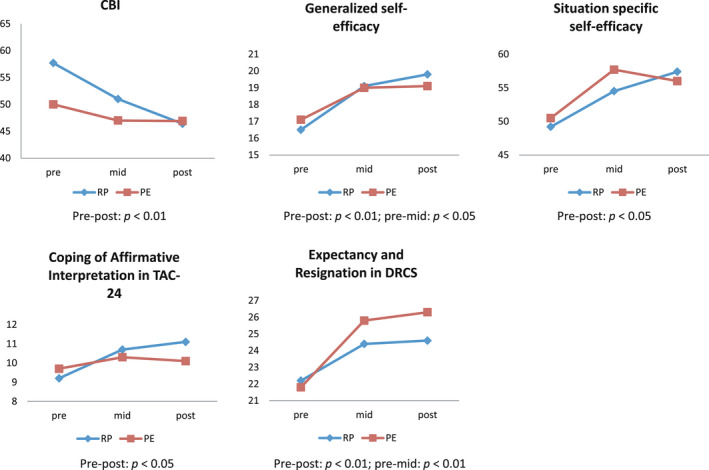
Mean score change of self‐rating scales at pre‐, mid‐, posttreatment in relapse prevention (RP) group and psychoeducation (PE) group: ITT analysis, no interaction in [time × group]. CBI, Coping Behaviors Inventory; DRCS, Drinking‐Related Cognitions Scale; TAC 24, Tri‐Axial Coping Scale

The GLM analysis showed no significant interaction in [time × group] in the score change of all scales. However, there was a significant main effect of [time] in the scores of CBI (*F*(1,46) = 7.53, *P* = .009), generalized self‐efficacy (*F*(1,46) = 10.16, *P* = .003), situation‐specific self‐efficacy (*F*(1,46) = 6.35, *P* = .015), coping of affirmative interpretation in TAC‐24 (*F*(1,46) = 4.74, *P* = .035), and the expectancy and resignation subscales of the DRCS (*F*(1,46) = 12.00, *P* = .001). The CBI score at posttreatment was significantly better than the pretreatment score (*P* = .008). The scores for generalized self‐efficacy, situation‐specific self‐efficacy, and expectancy and resignation of DRCS at mid‐treatment (*P* = .004) as well as posttreatment (*P* = .026) were significantly better than those at pretreatment (Figure 2). Also, in the subgroup analysis for male patients, there was no significant interaction in [time × group] in those scales.

## 4 DISCUSSION

This study was the first randomized controlled trial done as a pilot study to evaluate the efficacy of a CBT‐based relapse prevention (RP) program compared to psychoeducation (PE) for Japanese alcoholic patients. We did not find that the 12‐session RP program was superior to PE in terms of relapse rate at the 3‐ and 6‐month follow‐up periods. Also, although the self‐rating scales indicated significant psychological improvement between pre‐ and posttreatment, and pre‐ and mid‐treatment in both groups, there was no significant difference between them for change of score. Considering that the severity of alcohol use measured by the AUDIT at pretreatment was significantly greater in the PE group than in the RP group, the lack of superiority of the RP program cannot be attributed to the imbalance of the severity of alcohol abuse between the groups.

Our results were inconsistent with previous studies that showed the superiority of RP compared to psychoeducation in treating alcoholic patients. Miller and Wilbourne^3^ reported that among psychosocial treatments, evidence of efficacy was the strongest for social skills training and was weakest for education. Another meta‐analytic review reported that RP demonstrated moderate efficacy compared to psychoeducation.^7^

The inconsistency between our results and these previous studies may be accounted for by the following points. First, we replaced once‐a‐week psychoeducation sessions with once‐a‐week RP sessions in our inpatient treatment program, which itself consisted of multiple programs. Carroll^23^ reported that RP appeared to be effective relative to no‐treatment control, and equally as effective as other active treatments. Although a meta‐analysis of treatment for use of alcohol and illicit drugs showed a large effect size for CBT compared to no treatment, the effect size for CBT plus psychosocial treatment compared to psychosocial treatment alone was negative and insignificant.^8^ Ito et al^24^ pointed out that the reason for the lack of significant treatment effect in their study was that their inpatient treatment program might have been successful to the point that it left little room for additional improvement with the addition of the RP component. Similar to these studies, we also compared RP to active treatment (psychoeducation) and the participants received other types of interventions in the inpatient programs. This may have resulted in no significant difference between RP and PE.

Second, the results could be accounted for by cultural differences between Japan and Western countries. RP‐based treatment has not been empirically tested outside the United States, and even in the United States, it has been predominantly used for patients with mainstream cultural backgrounds.^9^ Cultural and/or social factors may influence treatment outcomes. These factors may include a worldview and value system (eg, individualistic vs collectivistic), culturally appropriate coping skills, alcohol expectancies, and available resources. RP was developed in the United States and the majority of clinical trials of treatment for alcohol use disorders were conducted in the United States and other Western countries, and there are only a few randomized controlled studies evaluating the efficacy of RP in Asian countries.^3^ Thus, further studies are necessary to evaluate the effectiveness of RP in Japan.

Notably, we found significant improvement even at the mid‐treatment point (after the sixth session) in both groups. This finding may suggest that a 6‐week inpatient program would be of some benefit for alcoholics. Although a randomized controlled study of RP compared to a 12‐step aftercare program showed that the number of RP sessions was correlated to better outcomes in drug use,^25^ a meta‐analysis indicated that the number of treatment sessions was negatively associated with the effect size.^8^ Another study, which evaluated the effectiveness of an inpatient group CBT program for alcohol dependence, showed that the attendance rate of CBT group sessions was not associated with improvement.^26^ In Japan, the inpatient period for treatment of alcoholism is usually 3 months. If short‐term treatment produces the same effect as longer treatment, short‐term treatment would be more cost‐effective and may increase motivation for inpatient treatment.

This study includes several limitations. First, by chance, there was a significant difference in the gender proportion between groups. The number of women in the RP group was very small compared to the PE group (1 vs 12). However, in the subgroup analysis of male patients, we did not find any difference in outcomes between the groups. A meta‐analysis has suggested that women appear to obtain more benefit from CBT than men do.^8^ Therefore, our study may suggest that the effectiveness of RP and PE is comparable in men although it is inconclusive in women.

Second, the primary outcome in our study was the relapse rate and we did not evaluate any other drinking outcomes. Bennett et al^27^ failed to detect a significant reduction of recurrence of any drinking as the treatment effect of RP. However, they detected clinically worthwhile improvements in other forms of drinking outcomes including occurrence of any heavy drinking and the frequency and amount of drinking. Even though our findings showed no significant difference between the groups in relapse rate at 3 and 6 months after discharge, there might be differences in other drinking outcomes. We expect further study to include these additional outcomes.

Third, the 6‐month follow‐up period may be too short to detect any preventive effects of RP. Previous RP intervention studies found no differential outcomes at earlier follow‐up, but results favored RP at 9, 12, and 15 months after discharge.^28^, ^29^, ^30^

Fourth, this study is a pilot with a small sample size. Generally, the required sample size is inversely proportional to the effect size.^31^ If the effect size of CBT for alcohol is small, a larger sample size is necessary to detect a treatment effect.

Fifth, we did not collect data on medication or follow‐up interventions, which are important variables that could influence outcomes. Since participants were randomized, these variables should theoretically be equal between the groups. However, we could not confirm if there was actually no between group difference.

## 5 CONCLUSIONS

A relapse prevention program did not show any better outcome than psychoeducation in our 12‐week inpatient program for alcohol dependence, although patients in both arms had significantly improved psychological outcomes. This study is the first randomized controlled trail on a pilot basis to evaluate the effectiveness of RP for alcoholic patients in Japan. Future research is expected to determine the effectiveness of RP for treatment of alcoholism in Japan with a larger sample size and a longer follow‐up period.

## CONFLICT OF INTEREST

The authors declare that they have no competing interests.

## AUTHOR CONTRIBUTIONS

TH and YA conceived of, designed, and performed the study. TH and YA supervised the treatment program and the outcome measurements. TH and YA drafted the manuscript and equally contributed for the study. MT, YY, and MU conducted the treatment programs and followed up the participants. YH performed the data management and statistical analysis. SO carried out the random assignment of the participants. NA participated in the design and coordination of the study, supervised the data analysis, and helped to draft the manuscript.

## APPROVAL OF THE RESEARCH PROTOCOL BY AN INSTITUTIONAL REVIEW BOARD

This study was approved by the Institutional Review Boards of the Tokyo Metropolitan Matsuzawa Hospital and the Tokyo Metropolitan Institute of Medical Science (approval number H23‐03).

## INFORMED CONSENT

All study participants provided informed consent.

## Data Availability

The data that support the findings of this study are openly available in “figshare” at https://doi.org/10.6084/m9.figshare.19314077.v1.
